# Characterization of dengue cases in a family medicine unit in Mexico

**DOI:** 10.17843/rpmesp.2026.431.14939

**Published:** 2026-03-30

**Authors:** Dayli Mancilla-Pérez, Gabriela García-Morales, Cinthya Jennifer Rayón-Castañeda, Juan Manuel Sánchez-Rebolledo, Ricardo Gil-Ojeda, Josue Rosaliano-Salinas

**Affiliations:** 1 Instituto Mexicano del Seguro Social, Ciudad de Mexico, Mexico.; 2 Universidad Autónoma de Guerrero, Guerrero, Mexico.

**Keywords:** Dengue, Severe Dengue, Dengue Virus, primary health care, Epidemiological Surveillance

## Abstract

The objective of this study was to describe the epidemiological and clinical characteristics of dengue cases treated in a family medicine unit in Guerrero, Mexico. A case series was conducted including patients diagnosed with dengue between January and December 2023; out of a total of 596 cases, 91.78% presented dengue without warning signs, 8.05% with warning signs, and 0.17% severe dengue. 2.7% of patients required hospitalization, and a case-fatality rate of 0.2% was recorded. The most frequent symptoms were fever (98.2%), headache (79.7%), and myalgia (75.3%). Dengue serology was performed in 28.1% of cases, with a positivity rate of 37%. The predominant clinical presentation observed in this research supports its utility in clinical practice for timely diagnosis and therapeutic decision-making according to the severity of the case; likewise, it highlights the importance of monitoring these patients for the early identification of warning signs.

## INTRODUCTION

Dengue is present in 120 countries, being endemic to subtropical and tropical regions of Asia, the Eastern Mediterranean, Africa, and the Americas ^1^. Approximately two-fifths of the world's population have suffered from dengue, with 390 million people diagnosed per year, 500,000 hospitalized, and 20,000 deaths due to complications of this disease ^2^.

The dengue virus (DENV) has four serotypes; of these, DENV-2 is the one that predominates in many countries, followed by serotypes 1 and 3, while DENV-4 is significantly lower than the rest ^3^. Currently, all four serotypes are circulating in Mexico ^4^.

In 2022, the majority of cases were reported in Brazil, Vietnam, the Philippines, Indonesia, and India ^5^; meanwhile, in Mexico, at the end of epidemiological week 49 of 2023, 260 cases per 100,000 inhabitants were recorded, with a total of 235,616 cases. Of these, 1,272 were severe dengue and 203 deaths occurred, with a case fatality rate of 0.86%; the most affected group was the 10 to 14-year-old age range, with a predominance in women ^6^.

The State of Guerrero in Mexico is one of the states most affected by dengue in recent years. One of the municipalities with the greatest impact was Acapulco de Juárez, with 32% of the reported dengue cases and an incidence rate of 31.2 per 100,000 inhabitants in 2021, showing an increase in incidence to 72.4 per 100,000 inhabitants in 2023 ^6^^,^^7^. Family Medicine Unit No. 26 is located in an endemic area for dengue, characterized by social vulnerability and limitations in continuous access to basic services, which favors virus transmission ^8^. Despite prevention and control efforts, dengue remains a relevant health concern in Mexico, Guerrero, and Acapulco, with a significant impact on public health and the local economy; therefore, the objective of this research was to describe the epidemiological and clinical characteristics of individuals treated for dengue from January to December 2023.

**KEY MESSAGES**

Motivation for the study. Despite sustained efforts in prevention and control, dengue continues to represent a major public health problem in Mexico.

Main findings. Of 596 cases analyzed, 91.78% presented with dengue without warning signs, 8.05% with warning signs, and 0.17% with severe dengue. Hospitalization was required for 2.7% of patients, and a case fatality rate of 0.2% was recorded. The most frequent symptoms were fever (98.2%), headache (79.7%), and myalgia (75.3%).

Implications. The clinical findings support their utility for timely diagnosis and therapeutic decision-making. Furthermore, they highlight the importance of population education for the early identification of warning signs.

## THE STUDY

### Design and population

An observational, descriptive case series study was conducted in the user population of a first-level health care unit, located in the suburban area of the municipality of Acapulco de Juárez, in the State of Guerrero, Mexico. A total of 596 files with a clinical diagnosis of dengue recorded in the Family Medicine Information System (SIMF) were reviewed.

The selection of cases was non-probabilistic by convenience, including all cases registered with a diagnosis of dengue during the study period; therefore, no sample size calculation was performed.

### Selection criteria

**Figure 1:**
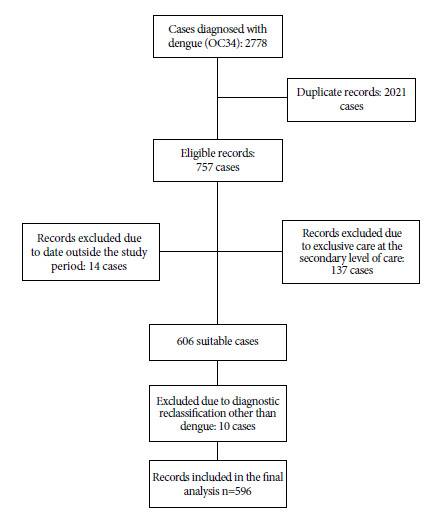
Flowchart of dengue case selection in a family medicine unit in Guerrero, Mexico.

Users of Family Medicine Unit No. 26 from January to December 2023 with a diagnosis of dengue without warning signs, dengue with warning signs, and severe dengue were included; of both sexes, any age, and possessing an electronic clinical record in the SIMF. Cases with a diagnosis of dengue treated exclusively at the second level of care and cases whose final diagnosis was different from dengue in subsequent notes of the electronic clinical record were excluded (Figure 1).

### Procedures

A total of 2,778 cases with a diagnosis of dengue were obtained from the diagnosis report of the SIMF local network of the family medicine unit. From the final report, the social security number was obtained to search for information contained in the SIMF medical notes, the epidemiological study concentrated in the National Epidemiological Surveillance System (SINAVE), and the laboratory reports of the person with dengue. Patients requiring hospitalization at another level of care were followed until discharge through the review of the electronic clinical record on the digital health ecosystem hospitalization platform. Data collection was carried out from August to November 2024. The obtained data were entered into an Excel sheet containing all the variables to be studied.

The collection instrument was a form designed by the researchers to record information, based on the epidemiological study questionnaire for suspected dengue cases used by all national health institutions in Mexico. Since it was a format designed exclusively for the extraction of clinical, epidemiological, and laboratory variables from clinical records, no validation process or pilot test was conducted. The template included two sections: sociodemographic information and clinical-epidemiological information. The presence of missing data, particularly in serological and blood count variables, was due to the retrospective nature of the study and the limited availability of such records in primary sources.

### Variables

Sociodemographic variables were age, sex, occupation, and schooling based on what was reported in the SIMF and SINAVE. Clinical-epidemiological information collected, such as systolic and diastolic blood pressure figures, was obtained from the vital signs section of the medical note in mm Hg. The variables of fever, nausea, vomiting, rash, headache, retro-orbital pain, myalgia, arthralgia, petechiae, epistaxis, abdominal pain, positive tourniquet test, hepatomegaly >2 cm, irritability, lethargy, and confusion were categorized as present when recorded in the medical note and absent when not mentioned. The severity of dengue was classified according to the standards established by the World Health Organization.

History of comorbidities such as diabetes mellitus, arterial hypertension, obesity, malnutrition, breastfeeding, pregnancy, human immunodeficiency virus (HIV), and asthma were obtained from each patient's history and electronic clinical record.

Hemoglobin in g/dL, hematocrit in percentage, leukocytes 10³/L, and platelets 10³/L were obtained from subsequent medical care notes and corroborated on the medical unit's laboratory platform from the complete blood count report. The researcher calculated the hematocrit/hemoglobin ratio as a criterion for suspected plasma leakage, considering it normal if less than or equal to 3.1, suggestive if 3.2 to 3.4, and indicative if 3.5 or higher. Leukopenia was considered less than 4.4 10³/L, leukocytosis greater than 11.3 10³/L, and normal from 4.4 to 11.3 10³/L; for platelets, normal was 150 to 450 10³/L, thrombocytopenia less than 150 10³/L, and thrombocytosis greater than 450 10³/L ^9^.

Hospitalization and death variables were collected from medical notes in the electronic clinical record of the second level of care in the emergency and hospitalization area. We considered hospitalization as admission to a hospital to receive medical or surgical assistance involving at least one overnight stay or having an assigned bed. Death was obtained from both medical notes and what was reported on the SINAVE platform; if the above was not reported, it was considered absent.

Serology for dengue virus was collected from SINAVE for DENV1, DENV2, DENV3, and DENV4, and in the absence of a record, it was coded as absent.

Due to the retrospective design of the study, some variables had incomplete data; analyses were performed with the available information, and missing data imputation techniques were not applied. The number of available data points varied according to the variable analyzed; missing data are indicated in each table as "no recorded data," and the sample size corresponds to the number of patients with available information for each variable.

### Statistical analysis

Data were imported from Excel to the statistical package SPSS V23 to perform the statistical analysis of the information. Univariate analyses were performed to obtain simple frequencies and proportions for qualitative variables; meanwhile, for quantitative variables, measures of central tendency and dispersion were obtained after checking for normality.

### Ethical aspects

The research was carried out with the prior acceptance and authorization of Local Research Committee 1101 and Ethics and Research Committee 11018 of the Mexican Social Security Institute, located in the state of Guerrero, Mexico, which is endorsed by the National Bioethics Commission. The research registration number was R-2024-1101-018.

## RESULTS

A total of 596 cases that met the selection criteria were registered; of these, 55.7% (332/596) were men, with an average age of 21 ± 14 years. Among the most frequent comorbidities, obesity (11.9%; 71/596), diabetes mellitus (2.7%; 16/596), and arterial hypertension (2.3%; 14/596) were identified. Sociodemographic characteristics and comorbidities are presented in table 1.

**Table 1:** Sociodemographic characteristics and comorbidities of patients with dengue treated at a family medicine unit in Acapulco, Guerrero, 2023 (n=596).

Variable		Dengue		DWWS		DW		SD	
		n	%	n	%	n	%	n	%
Sex									
	Male	332	55.7	305	91.9	26	7,8	1	0.3
	Female	264	44.3	242	91.7	22	8,3	0	0
Age									
	0-5 years	58	9.7	53	91.4	5	8,6	0	0
	6-11 years	118	19.8	107	90.7	10	8,5	1	0.8
	12-17 years	116	19.4	102	87.9	14	12,1	0	0
	18-26 years	120	20.1	112	93.3	8	6,7	0	0
	27-59 years	160	26.8	160	93.6	11	6,4	0	0
	60 years or older	13	2.1	13	100	0	0	0	0
Ocupation									
	Student	184	30.9	167	90.8	16	8,7	1	0-5
	Housewife	6	1.0	6	100	0	0	0	0
	Hotel employee	4	0.7	4	100	0	0	0	0
	Construction employee	1	0.2	1	100	0	0	0	0
	Healthcare employee	10	1.7	9	90.0	1	10	0	0
	Production services employee	7	1.2	7	100	0	0	0	0
	Retired or pensioned	1	0.2	1	100	0	0	0	0
	Others	130	21.8	122	93.8	8	6,2	0	0
	No recorded data	253	42.4	-	-	-	-	-	-
Schooling									
	None	7	1.2	6	85.7	1	14,3	0	0
	Able to read and write	88	14.8	83	94.3	5	5,7	0	0
	Primary school	34	5.7	28	82.4	6	17,6	0	0
	Secondary school	32	5.4	30	93.7	2	6,3	0	0
	High school	6	1.0	5	83.3	1	16,7	0	0
	Bachelor´s degree	7	1.2	6	85.7	1	14,3	0	0
	No data	422	70.8	-	-	--	-	-	-
Diabetes mellitus									
	Yes	16	2.7	15	93.7	1	6,3	0	0
	No	580	97.3	532	91.8	47	8	1	0.2
Hypertension									
	Yes	14	2.3	13	92.9	1	7,1	0	0
	No	582	97.7	534	91.8	47	8	1	0.2
Obesity									
	Yes	71	11.9	67	94.4	4	5,6	0	0
	No	298	50.0	274	91.9	24	8,1	0	0
	No data	227	38.1	-	-	-	-	-	-
Malnutrition									
	Yes	0	0	0	0	0	0	0	0
	No	567	95.1	521	91.9	45	7,9	1	0.2
	No data	29	4.9	-	-	-	-	-	-
Receiving breastfeeding									
	Yes	0	0	0	0	0	0	0	0
	No	596	100	547	91.8	48	8	1	0.2
Pregnancy									
	Yes	2	0.3	2	100	0	0	0	0
	No	594	99.7	545	91.8	48	8	1	0.2
HIV									
	Yes	0	0	0	0	0	0	0	0
	No	596	100	547	91.8	48	8	1	0.2
Asthma									
	Yes	2	0.3	2	100	0	0	0	0
	No	594	99.7	545	91.8	48	8	1	0.2

DWWS: Dengue without warning signs, DW: Dengue with warning signs, SD: Severe dengue

The predominant symptoms were fever (98.2%; 585/596), headache (79.7%; 475/596), and myalgia (75.3%; 449/596). The complete detail of symptoms is shown in table 2.

**Table 2:** Clinical symptoms present in patients with dengue treated at a family medicine unit in Guerrero, Mexico, 2023 (n=596).

		n	%
Fever			
	Yes	585	98.2
	No	11	1.8
Nauseas			
	Yes	318	53.4
	No	278	46.6
Vomiting			
	Yes	175	29.4
	No	421	70.6
Rash			
	Yes	30	5.0
	No	566	95.0
Headache			
	Yes	475	79.7
	No	121	20.3
Retro-orbital pain			
	Yes	329	55.2
	No	267	44.8
Myalgia			
	Yes	449	75.3
	No	147	24.7
Arthralgia			
	Yes	404	67.8
	No	192	32.2
Petechiae			
	Yes	16	2.7
	No	580	97.3
Epistaxis			
	Yes	3	0.5
	No	593	99.5
Abdominal pain			
	Yes	202	33.9
	No	394	66.1
Positive tourniquet test			
	Yes	5	0.8
	No	591	99.2
Hepatomegaly >2 cm			
	Yes	1	0.2
	No	595	99.8
Irritability			
	Yes	24	4.0
	No	572	96.0
Lethargy			
	Yes	7	1.2
	No	589	98.8
Confusion			
	Yes	1	0.2
	No	595	99.8

Dengue serology was performed in 28.1% (227/596) of cases, with 37% (84/227) positive results. Regarding viral typing, DENV-1 was identified in 5.3% (12/227), DENV-2 in 3.0% (9/227), and DENV-3 in 1.8% (4/227); in the remaining 88.9% (202/227), the serotype was not reported.

The complete blood count was performed in 491 patients. The main findings were leukopenia in 27.3% (134/491) and thrombocytopenia in 27.3% (134/491). A hematocrit/hemoglobin ratio ≥ 3.5 was observed in 1.2% of cases (6/491) (table 3).

**Table 3:** Hematological alterations in patients with dengue, Acapulco, 2023 (n=491).

		N	%
Leukocytes			
	Normal value	323	65.8
	Leukopenia	134	27.3
	Leukocytosis	34	6.9
Platelets			
	Normal value	353	71.9
	Thrombocytopenia	134	27.3
	Trombocytosis	4	0.8
Hematocrit/ hemoglobin ratio			
	Normal value	139	28.3
	Suggestive	346	70.5
	Indicative of hemoconcentration	6	1.2
Laboratory finding		Mean (SD)	
	Hemoglobin	13.7 (1.0)	
	Hematocrit	42.7 (5.0)	
	Hemoconcentration	3.1 (0.3)	
	Leukocytes	6.4 (3.0)	
	Platelets	196.0 (79.0)	

SD: Standard deviation

Regarding clinical classification, 91.8% (547/596) were diagnosed with dengue without warning signs, 8.0% (48/596) with dengue with warning signs, and 0.2% (1/596) with severe dengue. Hospitalization was required in 2.7% of cases (16/596), mainly in patients with severe dengue and some with warning signs. One death was recorded (0.2%; 1/596).

The proportion of dengue with warning signs was higher in the 12 to 17-year-old group (12.1%), followed by the 0 to 5-year-old group (8.6%). The only case of severe dengue occurred in the 6 to 11-year-old age group.

## DISCUSSION

The findings showed that the predominant symptoms were fever, headache, and myalgia; dengue serology was not performed on all patients, and one-third of these presented a positive result for dengue virus, where DENV-1 predominated. The main alteration in the blood count was thrombocytopenia, and regarding severity, dengue without warning signs prevailed.

The predominant symptoms in the analyzed dengue cases were fever, accompanied by headache, myalgias, and arthralgias; this is consistent with reports from other studies conducted in endemic countries such as Peru and Cuba, and guidelines issued by the Pan American Health Organization ^10^^,^^11^. The similarity with previous studies highlights the clinical presentation of dengue in different geographical contexts and reinforces the validity of the diagnostic criteria issued by the WHO.

The WHO classification is useful for stratifying severity and guiding clinical decision-making at different levels of care. In the present research, dengue without warning signs predominated; these results differ from those reported by Taylor et al., where 82.5% of hospitalized patients presented at least one warning sign and 2.8% developed severe dengue ^12^. Meanwhile, in a prospective multicenter study by Yacoub et al., the distribution based on severity was 43% without warning signs, 52% with warning signs, and 5% severe ^13^. Dengue without warning signs predominates in research including the general population or first-level healthcare units; whereas in studies conducted in hospital settings, dengue with warning signs occurs more frequently. The present study excluded patients treated exclusively at the second level of care, which underestimated the real frequency of severe cases. Nevertheless, clinical staff must keep warning signs in mind at all times regardless of the level of medical care, in order to act timely and refer patients to emergency services.

Age can be a relevant factor in dengue severity; in this research, the cases of dengue with warning signs and severe dengue were 8.3%; a figure lower than that reported by Rosso F et al., who found 43% of cases with warning signs and 22% with severe dengue in an elderly population hospitalized in Colombia. In another study conducted in the State of Guerrero, Mexico, by Alvarado-Castro et al., cases with warning signs were 58% in a pediatric population of a second-level hospital. These differences can be explained by several reasons: the aforementioned studies included vulnerable populations (elderly and hospitalized pediatric population), while the present study included patients of all ages treated at the medical unit; furthermore, variations in viral circulation and the epidemiological timing of the outbreak also influence the proportion of severe cases ^14^^,^^15^. Our results agree with those reported in Brazil by Teixeira et al., where by including patients of all ages, the occurrence of dengue with warning signs and severe dengue was 3.5%; that is, the incidence of severe cases is lower when the sample is composed of different age groups compared to when extreme ages are included, such as pediatric and elderly populations ^16^.

In our research, the predominant serotype was DENV-1; however, these results regarding viral circulation cannot be extrapolated to the rest of the population because serology for dengue was only performed on one-third of the patients. Other studies have reported a predominance of other serotypes; in a study conducted in 2023 in different regions of Mexico, a higher occurrence of DENV-3 was identified ^17^; also in Mexico, a study was conducted from January to December 2021 where a predominance of DENV-2 was observed at 31.2% ^18^; both studies report that DENV-4 has the lowest occurrence. However, in Mexico, the serotyping of dengue in 2023 was DENV-3 (59.3%), DENV-2 (21.8%), DENV-1 (16.8%), and DENV-4 (2.1%) ^6^.

Regarding laboratory tests, the findings for the hematocrit/hemoglobin ratio, leukopenia, and thrombocytopenia observed in this study are consistent with those reported by Matta et al., who identified that most patients with dengue presented mild thrombocytopenia and leukopenia. These parameters are well known as indicators of disease progression and correlate with a higher risk of severe complications, such as severe dengue, which reinforces the utility of the complete blood count as a basic study to be performed in a person with a dengue diagnosis to assess the severity of the disease ^19^^,^^20^.

The results in this study are consistent with the literature in terms of predominant clinical symptoms, the distribution of dengue serotypes, and hematological parameters associated with disease severity; however, variations in the frequency of severe cases and serotype distribution among different age groups reflect the heterogeneity of the disease and the need for a contextualized approach to its management. One of the main strengths of this study is the application of the dengue classification proposed by the WHO, based on the severity of the disease and the need for clinical management; this classification allows for a more precise stratification of patients and better guidance in clinical decision-making. Another strength of this research is that it was conducted in an endemic area for dengue, which allows the findings to more accurately reflect the dynamics of the disease in a context of high incidence, providing valuable information for epidemiological surveillance and public health decision-making.

Among the main limitations of the study is its design as a case series with non-probabilistic convenience sampling, which significantly limits the external validity of the results and prevents their generalization to other populations. This type of design only allows for describing the frequency of clinical and epidemiological characteristics observed but not establishing associations or causal relationships between the variables studied. Likewise, the exclusion of patients treated exclusively at the second level of care introduces a possible selection bias, as it may underestimate the real frequency of severe dengue cases and hospitalizations. Being a retrospective study based on clinical records, there was an absence of information for some variables, mainly in the performance of serological tests and laboratory studies.

Finally, since there was no comparison group or longitudinal follow-up, the results should be interpreted only as a description of the clinical behavior of dengue in a first-level care unit. It is recommended to conduct prospective research, where cases are entered into the study from diagnosis and given timely follow-up for each variable. It is also recommended to collect other social factors that may be involved in the progression to severe dengue ^21^.

The predominant clinical presentation, such as fever accompanied by headache, myalgia, and arthralgia, should guide clinical staff toward a diagnosis, with the goal of establishing management based on severity and being alert to warning signs during the course of the disease to avoid complications or deaths. Therefore, the results of this research provide local epidemiological and clinical information that allows for strengthening epidemiological surveillance, optimizing the timely detection of warning signs at the first level of care, and contributing to the prevention of severe complications and mortality from dengue in the region. However, areas of opportunity were identified in the medical care provided to persons diagnosed with dengue, such as the absence of serological confirmation, the lack of reporting of laboratory studies, and the failure to record all clinical data.
