# Supplementary material for: Characterization of dengue cases in a family medicine unit in Mexico
Source: Rev Peru Med Exp Salud Publica. 2026 Mar 30;43(1):90–7. doi: 10.17843/rpmesp.2026.431.14939 (PMC13245988; doi:10.17843/rpmesp.2026.431.14939)
Supplement: Translation

# Translation: Caracterización de los casos de dengue en una unidad de medicina familiar en México

**Authors:** Dayli Mancilla-Pérez, Gabriela García-Morales, Cinthya Jennifer Rayón-Castañeda, Juan Manuel Sánchez-Rebolledo, Ricardo Gil-Ojeda, Josue Rosaliano-Salinas

**Affiliations:** 1 Instituto Mexicano del Seguro Social, México.; 2 Universidad Autónoma de Guerrero, Guerrero, México.

**Keywords:** Dengue, Dengue Grave, Virus del Dengue, Atención Primaria de Salud, Vigilancia Epidemiológica

## Abstract

El objetivo de este estudio fue describir las características epidemiológicas y clínicas de los casos de dengue atendidos en una unidad de medicina familiar en Guerrero, México. Se realizó una serie de casos que incluyó pacientes diagnosticados con dengue entre enero y diciembre de 2023; de un total de 596 casos, el el 91,78% presentó dengue sin signos de alarma, el 8,05% con signos de alarma y el 0,17% dengue grave. El 2,7% de los pacientes requirió hospitalización y se registró una letalidad del 0,2%. Los síntomas más frecuentes fueron fiebre (98,2%), cefalea (79,7%) y mialgias (75,3%). Se realizó serología para dengue en el 28,1% de los casos, con una positividad del 37%. La sintomatología predominante observada en esta investigación respalda su utilidad en la práctica clínica para el diagnóstico oportuno y la toma de decisiones terapéuticas según la gravedad del cuadro; asimismo, resalta la importancia del seguimiento de estos pacientes para la identificación temprana de datos de alarma.

## INTRODUCCIÓN

El dengue está presente en 120 países, siendo endémico de regiones subtropicales y tropicales de la región de Asia, el Mediterráneo Oriental, África y América ^1^. Aproximadamente dos quintas partes de la población mundial han padecido de dengue, donde 390 millones de personas se diagnostican por año, 500 000 se hospitalizan y 20 000 mueren por complicaciones de esta enfermedad ^2^. El virus del dengue (DENV) tiene cuatro serotipos; de estos el DENV-2 es el que predomina en gran parte de los países, seguido de los serotipos 1 y 3, el DENV-4 es significativamente menor que el resto ^3^. En la actualidad, en México se encuentran circulando los cuatro serotipos ^4^.

En 2022, la mayoría de los casos se notificaron en Brasil, Vietnam, Filipinas, Indonesia e India ^5^; en tanto, en México al cierre de la semana epidemiológica 49 de 2023 se registraron 260 casos por 100 000 habitantes, con un total de 235 616 casos, de estos 1272 fueron dengue grave y 203 defunciones con una tasa de letalidad de 0,86%; siendo el grupo con mayor afectación el de 10 a 14 años y con predominio en mujeres ^6^.

El Estado de Guerrero en México, es uno de los estados más afectados por dengue en los últimos años, donde uno de los municipios con mayor afectación fue Acapulco de Juárez con el 32% de los casos reportados de dengue y una tasa de incidencia de 31,2 por cada 100 000 habitantes en el año 2021, con un aumento en la incidencia de 72,4 por cada 100 000 habitantes en 2023 ^6^^,^^7^. La Unidad de Medicina Familiar No. 26 se localiza en una zona endémica para dengue, con condiciones de vulnerabilidad social y limitaciones en el acceso continuo a servicios básicos, lo que favorece la transmisión del virus ^8^. A pesar de los esfuerzos de prevención y control, el dengue sigue siendo una preocupación de salud relevante en México, Guerrero, Acapulco, con impacto significativo en la salud pública y la economía local; por lo que el objetivo de esta investigación fue describir las características epidemiológicas y clínicas de las personas atendidas por dengue en el periodo enero a diciembre 2023.

**MENSAJE CLAVE**

Motivación para realizar el estudio. A pesar de los esfuerzos sostenidos en prevención y control, el dengue continúa representando un importante problema de salud pública en México.

Principales hallazgos. De 596 casos analizados, eel 91,78% presentó dengue sin signos de alarma, el 8,05% con signos de alarma y el 0,17% dengue grave. El 2,7% de los pacientes requirió hospitalización y se registró una letalidad del 0,2%. Los síntomas más frecuentes fueron fiebre (98,2%), cefalea (79,7%) y mialgias (75,3%).

Implicancias. Los hallazgos clínicos respaldan su utilidad para el diagnóstico oportuno y la toma de decisiones terapéuticas. Asimismo, destacan la importancia de la educación de la población para la identificación temprana de signos de alarma.

## EL ESTUDIO

### Diseño y población

Se realizó un estudio observacional, descriptivo de serie de casos en población usuaria de una unidad médica de primer nivel de atención, la cual se ubica en zona conurbana del municipio de Acapulco de Juárez, en el Estado de Guerrero, México. Se revisaron 596 expedientes con registro diagnóstico de dengue por clínica en el sistema de información de medicina familiar (SIMF).

La selección de los casos fue no probabilística por conveniencia, con ingreso de la totalidad de los casos registrados con diagnóstico de dengue durante el periodo de estudio, por lo que no se realizó cálculo de tamaño de muestra.

### Criterios de selección

Se incluyó a usuarios de la unidad de medicina familiar No. 26 de enero a diciembre de 2023 con diagnóstico de dengue sin signos de alarma, dengue con signos de alarma y dengue severo; de ambos sexos, de cualquier edad y que contaran con expediente clínico electrónico en el SIMF; se excluyó a casos con diagnóstico de dengue tratados de forma exclusiva en segundo nivel de atención y los casos cuyo diagnóstico final fue diferente al de dengue en notas subsecuentes del expediente clínico electrónico figura 1.

**Figura 1:** Flujograma de seleccion de casos de dengue en una unidad de medicina familiar en Guerrero, Mexico.

### Procedimientos

Se obtuvieron 2778 casos con diagnóstico de dengue en el reporte de diagnósticos de la red local de la unidad de medicina familiar del SIMF, del reporte final se obtuvo el número de seguridad social para buscar la información contenida en las notas médicas del SIMF, del estudio epidemiológico concentrado en el Sistema Nacional de Vigilancia Epidemiológica (SINAVE) y los reportes de laboratorio de la persona con dengue. Los pacientes que requirieron hospitalización en otro nivel de atención fueron seguidos hasta su egreso a través de la revisión del expediente clínico electrónico de la plataforma de hospitalización del ecosistema digital en salud. La recolección de los datos se llevó a acabo de agosto a noviembre de 2024. Los datos obtenidos se capturaron en una cédula de Excel que contenía la totalidad de las variables a estudiar.

El instrumento de recolección fue una cédula diseñada por los investigadores para registrar la información, basada en el cuestionario del estudio epidemiológico de caso sospechoso de dengue utilizado por todas las instituciones de salud a nivel nacional en México; dado que se trató de un formato diseñado exclusivamente para la extracción de variables clínicas, epidemiológicas y de laboratorio a partir de expedientes clínicos, no se realizó un proceso de validación ni prueba piloto. La plantilla incluyó dos apartados: información sociodemográfica e información clínico-epidemiológica. La presencia de datos faltantes, particularmente en variables serológicas y de biometría hemática, se debió a la naturaleza retrospectiva del estudio y a la disponibilidad limitada de dichos registros en las fuentes primarias.

### Variables

Las variables sociodemográficas fueron edad, sexo, ocupación, y escolaridad con base a lo reportado en el SIMF y SINAVE. La información clínico-epidemiológica recolectada como cifras de presión sistólica y diastólica se recolectó del apartado de signos vitales de la nota médica en mm Hg. Las variables de fiebre, náusea, vómito, exantema, cefalea, dolor retro orbital, mialgia, artralgia, petequias, epistaxis, dolor abdominal, prueba positiva a torniquete, hepatomegalia >2 cm, irritabilidad, letargo y confusión fueron categorizadas como presentes cuando se consignaron en la nota médica y ausentes cuando no se hizo mención. La gravedad de dengue se clasificó de acuerdo con lo establecido por la Organización Mundial de Salud.

Los antecedentes de comorbilidades como diabetes mellitus, hipertensión arterial, obesidad, desnutrición, lactancia materna, embarazo, virus de inmunodeficiencia humana (VIH) y asma se obtuvieron de la historia y expediente clínico electrónico de cada paciente.

La hemoglobina en g/dL, el hematocrito en porcentaje, leucocitos 10^3^/L y plaquetas 10^3^/L fueron obtenidos de las notas subsecuentes de atención médica y corroborados en la plataforma de laboratorio de la unidad médica del reporte de biometría hemática. El investigador realizó el cálculo de la relación hematocrito/hemoglobina como criterio de sospecha de fuga plasmática considerando como normal si es menor o igual a 3,1, sugestivo 3,2 a 3,4 e indicativo 3,5 o mayor. Se consideró como leucopenia menos de 4,4 10^3^/L, lecucocitosis mayor a 11,3 10^3^/L y normal de 4,4 a 11,3 10^3^/L; en plaquetas fue normal de 150 a 450 10^3^/L, plaquetopenia menos de 150 10^3^/L y trombocitosis mayor de 450 10^3^/L ^9^.

Las variables de hospitalización y fallecimiento se recabaron de notas médicas del expediente clínico electrónico de segundo nivel de atención del área de urgencias y hospitalización. Consideramos hospitalización como el ingreso a hospital para recibir asistencia médica o médico quirúrgico que suponga, al menos, pasar una noche en el mismo o tener una cama asignada. El fallecimiento se obtuvo tanto de las notas médicas como de lo reportado en la plataforma de SINAVE, en caso de no estar reportado lo anterior se consideró como ausente.

La serología para virus dengue fue recolectada del SINAVE en DENV1, DENV2, DENV3 y DENV4, y en ausencia de registro se codificó como ausente.

Debido al diseño retrospectivo del estudio, algunas variables presentaron datos incompletos; los análisis se realizaron con la información disponible y no se aplicaron técnicas de imputación de datos faltantes. El número de datos disponibles varió según la variable analizada, los datos faltantes se señalan en cada tabla como sin dato registrado, y el tamaño de muestra corresponde al número de pacientes con información disponible para cada variable.

### Análisis estadístico

Los datos se importaron desde Excel al paquete estadístico SPSS V23 para realizar el análisis estadístico de la información. Se realizó análisis univariados para obtener frecuencias simples y proporciones en las variables cualitativas; en tanto, para variables cuantitativas se obtuvieron medidas de tendencia central y de dispersión previa comprobación de la normalidad.

### Aspectos éticos

La investigación se llevó a cabo con previa aceptación y autorización de un Comité Local de Investigación 1101 y Comité de Ética e Investigación 11018 del Instituto Mexicano del Seguro Social, localizado en el estado de Guerrero, México, el cual está avalado por la Comisión Nacional de Bioética, con el número de registro de la investigación fue R-2024-1101-018.

## RESULTADOS

Se registraron 596 casos que cumplieron con los criterios de selección; de ellos, el 55,7% (332/596) correspondió a hombres, con una edad promedio de 21 ± 14 años. Entre las comorbilidades más frecuentes se identificaron obesidad (11,9%; 71/596), diabetes mellitus (2,7%; 16/596) e hipertensión arterial (2,3%; 14/596). Las características sociodemográficas y comorbilidades se presentan en la tabla 1.

**Tabla 1:** Características sociodemográficas y comorbilidades de los pacientes con dengue atendidos en una unidad de medicina familiar de Acapulco, Guerrero, 2023 (n=596).

Variable		Dengue		DSS		DCS		DG	
		n	%	n	%	n	%	n	%
Sexo									
	Hombre	332	55,7	305	91,9	26	7,8	1	0,3
	Mujer	264	44,3	242	91,7	22	8,3	0	0
Edad									
	0-5 años	58	9,7	53	91,4	5	8,6	0	0
	6-11 años	118	19,8	107	90,7	10	8,5	1	0,8
	12-17 años	116	19,4	102	87,9	14	12,1	0	0
	18-26 años	120	20,1	112	93,3	8	6,7	0	0
	27-59 años	160	26,8	160	93,6	11	6,4	0	0
	60 o más años	13	2,1	13	100	0	0	0	0
Ocupación									
	Estudiante	184	30,9	167	90,8	16	8,7	1	0,5
	Ama de casa	6	1,0	6	100	0	0	0	0
	Empleado hotelería	4	0,7	4	100	0	0	0	0
	Empleado construcción	1	0,2	1	100	0	0	0	0
	Empleado de salud	10	1,7	9	90,0	1	10	0	0
	Empleado de servicios de producción	7	1,2	7	100	0	0	0	0
	Jubilado o pensionado	1	0,2	1	100	0	0	0	0
	Otros	130	21,8	122	93,8	8	6,2	0	0
	Sin dato registrado	253	42,4	-	-	-	-	-	-
Escolaridad									
	Ninguna	7	1,2	6	85,7	1	14,3	0	0
	Sabe leer y escribir	88	14,8	83	94,3	5	5,7	0	0
	Primaria	34	5,7	28	82,4	6	17,6	0	0
	Secundaria	32	5,4	30	93,7	2	6,3	0	0
	Preparatoria	6	1,0	5	83,3	1	16,7	0	0
	Licenciatura	7	1,2	6	85,7	1	14,3	0	0
	Sin dato	422	70,8	-	-	--	-	-	-
Diabetes mellitus									
	Sí	16	2,7	15	93,7	1	6,3	0	0
	No	580	97,3	532	91,8	47	8	1	0,2
Hipertensión									
	Sí	14	2,3	13	92,9	1	7,1	0	0
	No	582	97,7	534	91,8	47	8	1	0,2
Obesidad									
	Sí	71	11,9	67	94,4	4	5,6	0	0
	No	298	50,0	274	91,9	24	8,1	0	0
	Sin dato	227	38,1	-	-	-	-	-	-
Desnutrición									
	Sí	0	0	0	0	0	0	0	0
	No	567	95,1	521	91,9	45	7,9	1	0,2
	Sin dato	29	4,9	-	-	-	-	-	-
Recibe lactancia materna									
	Sí	0	0	0	0	0	0	0	0
	No	596	100	547	91,8	48	8	1	0,2
Embarazo									
	Sí	2	0,3	2	100	0	0	0	0
	No	594	99,7	545	91,8	48	8	1	0,2
VIH									
	Sí	0	0	0	0	0	0	0	0
	No	596	100	547	91,8	48	8	1	0,2
Asma									
	Sí	2	0,3	2	100	0	0	0	0
	No	594	99,7	545	91,8	48	8	1	0,2

DSS: Dengue sin signos de alarma, DCS: Dengue con signos de alarma, DG: Dengue Grave

Los síntomas predominantes fueron fiebre (98,2%; 585/596), cefalea (79,7%; 475/596) y mialgias (75,3%; 449/596). El detalle completo de los síntomas se muestra en la tabla 2.

**Tabla 2:** Síntomas clínicos presentes en pacientes con dengue atendidos en una unidad de medicina familiar en Guerrero, México, 2023 (n=596).

		n	%
Fiebre			
	Sí	585	98,2
	No	11	1,8
Nauseas			
	Sí	318	53,4
	No	278	46,6
Vomito			
	Sí	175	29,4
	No	421	70,6
Exantema			
	Sí	30	5,0
	No	566	95,0
Cefalea			
	Sí	475	79,7
	No	121	20,3
Dolor retro-orbital			
	Sí	329	55,2
	No	267	44,8
Mialgia			
	Sí	449	75,3
	No	147	24,7
Artralgia			
	Sí	404	67,8
	No	192	32,2
Petequias			
	Sí	16	2,7
	No	580	97,3
Epistaxis			
	Sí	3	0,5
	No	593	99,5
Dolor abdominal			
	Sí	202	33,9
	No	394	66,1
Prueba positiva a torniquete			
	Sí	5	0,8
	No	591	99,2
Hepatomegalia >2 cm			
	Sí	1	0,2
	No	595	99,8
Irritabilidad			
	Sí	24	4,0
	No	572	96,0
Letargo			
	Sí	7	1,2
	No	589	98,8
Confusión			
	Sí	1	0,2
	No	595	99,8

Se realizó serología para dengue en el 28,1% (227/596) de los casos, con un 37% (84/227) de resultados positivos. En cuanto a la tipificación viral, se identificó DENV-1 en el 5,3% (12/227), DENV-2 en el 3,0% (9/227) y DENV-3 en el 1,8% (4/227); en el 88,9% (202/227) restante no se reportó el serotipo.

La biometría hemática se efectuó en 491 pacientes. Los principales hallazgos fueron leucopenia en el 27,3% (134/491) y trombocitopenia en el 27,3% (134/491). La relación hematocrito/hemoglobina ≥ 3,5 se observó en el 1,2% de los casos (6/491) (tabla 3).

**Tabla 3:** Alteraciones hematológicas en pacientes con dengue, Acapulco, 2023 (n=491).

		N	%
Leucocitos			
	Valor normal	323	65,8
	Leucopenia	134	27,3
	Leucocitosis	34	6,9
Plaquetas			
	Valor normal	353	71,9
	Plaquetopenia	134	27,3
	Trombocitosis	4	0,8
Relación hematocrito/hemoglobina			
	Valor normal	139	28,3
	Sugestiva	346	70,5
	Indicativa hemoconcentración	6	1,2
Hallazgo de laboratorio		Media (DE)	
	Hemoglobina	13,7 (1,0)	
	Hematocrito	42,7 (5,0)	
	Hemoconcentración	3,1 (0,3)	
	Leucocitos	6,4 (3,0)	
	Plaquetas	196,0 (79,0)	

DE: Desviación estándar

En cuanto a la clasificación clínica, el 91,8% (547/596) fue diagnosticado con dengue sin signos de alarma, el 8,0% (48/596) con dengue con signos de alarma y el 0,2% (1/596) con dengue grave. La hospitalización fue requerida en el 2,7% de los casos (16/596), principalmente en pacientes con dengue grave y algunos con signos de alarma. Se registró una defunción (0,2%; 1/596).

La proporción de dengue con signos de alarma fue mayor en el grupo de 12 a 17 años (12,1%), seguido del grupo de 0 a 5 años (8,6%). El único caso de dengue grave se presentó en el grupo etario de 6 a 11 años.

## DISCUSIÓN

Los hallazgos evidenciaron que los síntomas predominantes fueron fiebre, cefalea y mialgias; no se realizó serología para dengue a la totalidad de los pacientes, una tercera parte de estos presentó resultado positivo para virus dengue, donde predominó el DENV-1. La principal alteración en la biometría hemática fue la plaquetopenia, y con respecto a la gravedad prevaleció el dengue sin signos de alarma.

Los síntomas predominantes en los casos de dengue analizados fueron fiebre, acompañada de cefalea, mialgias y artralgias; lo cual, es consistente con lo reportado en otros estudios realizados en países endémicos como Perú, Cuba y lo emitido por la Organización Panamericana de la Salud ^10^^,^^11^. La similitud con estudios previos resalta la presentación clínica del dengue en diferentes contextos geográficos y refuerza la validez de los criterios diagnósticos emitidos por la OMS.

La clasificación de la OMS es útil para estratificar la severidad y guiar la toma de decisiones clínicas en distintos niveles de atención. En la presente investigación predominó el dengue sin signos de alarma; estos resultados difieren de lo reportado por Taylor y cols., donde el 82,5% de los pacientes hospitalizados presentó al menos un signo de alarma y el 2,8% desarrolló dengue grave ^12^. En tanto, un estudio multicéntrico prospectivo de Yacoub y cols., la distribución con base a la gravedad fue de 43% sin signos de alarma, 52% con signos de alarma y 5% severo ^13^. El dengue sin signos de alarma predomina en las investigaciones donde se incluyeron personas de población abierta o de unidades médicas de primer nivel de atención; mientras que, aquellas realizadas en contextos hospitalarios el dengue con signos de alarma es el de mayor ocurrencia. El presente estudio excluyó a los pacientes tratados de forma exclusiva en segundo nivel de atención, lo que subestimo la frecuencia real de los casos graves. No obstante, el personal clínico debe tener presente en todo momento los datos de alarma sin importar el nivel de atención médica, con la finalidad de actuar de manera oportuna y derivar a los servicios de urgencias a los pacientes.

La edad puede ser un factor relevante en la severidad del dengue, en esta investigación los casos de dengue con signos de alarma y grave fue de 8,3%; cifra menor a la reportada por Rosso F y cols., quienes encontraron un 43% de casos con signos de alarma y 22% con dengue grave en población adulta mayor hospitalizada en Colombia; en otra investigación realizada en el Estado Guerrero, México, por Alvarado-Castro y cols., los casos con signos de alarma fueron del 58% en población pediátrica de un hospital de segundo nivel de atención. Estas diferencias pueden explicarse por varias razones: en los estudios mencionados se incluyeron poblaciones vulnerables (adultos mayores y población pediátrica hospitalizada); mientras que, en el presente estudio se incluyeron pacientes de todas las edades atendidos en la unidad médica; además, las variaciones en la circulación viral y el momento epidemiológico del brote también influyen en la proporción de casos graves ^14^^,^^15^. Nuestros resultados concuerdan con lo reportado en Brasil por Teixeira y cols., donde al incluir pacientes de todas las edades, la ocurrencia de dengue con signos de alarma y grave fue de 3,5%; es decir, la incidencia de los casos graves es menor cuando la muestra se integra por diferentes grupos de edad, en comparación de cuando se incluyen edades extremas, como es el caso de la edad pediátrica y del adulto mayor ^16^.

En nuestra investigación el serotipo predominante fue el DENV-1; no obstante, estos resultados con relación a la circulación viral, no se pueden extrapolar al resto de la población debido a que solo se realizó la serología para dengue a una tercera parte de los pacientes. En otras investigaciones se ha reportado predominio de otros serotipos; en una investigación realizada en 2023 en diferentes regiones de México, se identificó mayor ocurrencia de DENV-3 ^17^; también en México, se llevó a cabo una investigación en el periodo de enero a diciembre de 2021 en donde se observó un predominio de DENV-2 con 31,2% ^18^; ambos estudios, reportan que el de menor ocurrencia es el DENV-4; sin embargo, en México la serotipificación de dengue en 2023 fue DENV-3 (59,3%), DENV-2 (21,8%), DENV-1 (16,8%) y DENV-4 (2,1%) ^6^.

En cuanto a los exámenes de laboratorio, los hallazgos de relación hematocrito/hemoglobina, leucopenia y plaquetopenia observados en este estudio son congruentes con los reportados por Matta *et al.*, quienes identificaron que la mayoría de los pacientes con dengue presentaban trombocitopenia leve y leucopenia. Estos parámetros son bien conocidos como indicadores de la progresión de la enfermedad y se correlacionan con un mayor riesgo de complicaciones graves, como el dengue grave, lo que refuerza la utilidad de la biometría hemática como un estudio básico a realizar en la persona con diagnóstico de dengue para valorar la gravedad de la enfermedad ^19^^,^^20^.

Los resultados en este estudio son consistentes con la literatura en términos de los síntomas clínicos predominantes, la distribución de los serotipos de dengue y los parámetros hematológicos asociados con la severidad de la enfermedad; sin embargo, las variaciones en la frecuencia de los casos graves y la distribución de los serotipos entre los diferentes grupos de edad reflejan la heterogeneidad de la enfermedad y la necesidad de un enfoque contextualizado para su manejo. Una de las principales fortalezas de este estudio es la aplicación de la clasificación del dengue propuesta por la OMS, basada en la gravedad de la enfermedad y la necesidad del manejo clínico, esta clasificación permite una estratificación más precisa de los pacientes y una mejor orientación en la toma de decisiones clínicas. Otra de las fortalezas de esta investigación es que fue realizada en una zona endémica para el dengue, esto permite que los hallazgos reflejen de manera más precisa la dinámica de la enfermedad en un contexto de alta incidencia, lo que aporta información valiosa para la vigilancia epidemiológica y la toma de decisiones en la salud pública.

Entre las principales limitaciones del estudio se encuentra su diseño como serie de casos con muestreo no probabilístico por conveniencia, lo cual limita de forma importante la validez externa de los resultados y evita su generalización a otras poblaciones. Este tipo de diseño únicamente permite describir la frecuencia de las características clínicas y epidemiológicas observadas, pero no establecer asociaciones ni relaciones causales entre las variables estudiadas. Asimismo, la exclusión de pacientes atendidos exclusivamente en segundo nivel de atención introduce un posible sesgo de selección, ya que puede subestimar la frecuencia real de los casos de dengue grave y de hospitalización. Al tratarse de un estudio retrospectivo basado en registros clínicos, hubo ausencia de información en algunas variables, principalmente en la realización de pruebas serológicas y estudios de laboratorio.

Finalmente, al no contar con un grupo de comparación ni con seguimiento longitudinal, los resultados deben interpretarse únicamente como una descripción del comportamiento clínico del dengue en una unidad de primer nivel de atención. Se aconseja realizar investigaciones del tipo prospectivo, donde los casos se ingresen a la investigación desde su diagnóstico y se les otorgue un seguimiento puntual de cada una de las variables. También es recomendable recolectar otros factores sociales que puedan estar involucrados en la evolución para un dengue grave ^21^.

La clínica predominante como la fiebre acompañada de cefalea, mialgias y artralgias, debe orientar al personal clínico al diagnóstico, con la finalidad de establecer un manejo con base a la gravedad y estar atentos a los signos de alarma durante la evolución de la enfermedad, para evitar complicaciones o fallecimientos. Por tanto, los resultados de esta investigación aportan información epidemiológica y clínica local que permite fortalecer la vigilancia epidemiológica, optimizar la detección oportuna de datos de alarma en el primer nivel de atención y contribuir a la prevención de complicaciones graves y mortalidad por dengue en la región. Sin embargo, se identificaron áreas de oportunidad en las atenciones médicas otorgadas a las personas con diagnóstico con dengue, como la ausencia de confirmación por serología, la ausencia de reporte de estudios de laboratorio y la falta de registro de todos los datos clínicos. No obstante, la clínica predominante como la fiebre acompañada de cefalea, mialgias y artralgias, debe orientar al personal clínico al diagnóstico, con la finalidad de establecer un manejo con base a la gravedad y estar atentos a los signos de alarma durante la evolución de la enfermedad, para evitar complicaciones o fallecimientos. Por tanto, los resultados de esta investigación aportan información epidemiológica y clínica local que permite fortalecer la vigilancia epidemiológica, optimizar la detección oportuna de datos de alarma en el primer nivel de atención y contribuir a la prevención de complicaciones graves y mortalidad por dengue en la región.
